# SARS-CoV-2 Infection and Alpha-Synucleinopathies: Potential Links and Underlying Mechanisms

**DOI:** 10.3390/ijms252212079

**Published:** 2024-11-10

**Authors:** Joanna Agata Motyl, Grażyna Gromadzka, Grzegorz Arkadiusz Czapski, Agata Adamczyk

**Affiliations:** 1 Department of Hybrid Microbiosystems Engineering, Nalecz Institute of Biocybernetics and Biomedical Engineering, Polish Academy of Sciences, Ks. Trojdena 4 St., 02-109 Warsaw, Poland; jmotyl@ibib.waw.pl; 2 Department of Biomedical Sciences, Faculty of Medicine, Collegium Medicum, Cardinal Stefan Wyszynski University, Wóycickiego 1/3, 01-938 Warsaw, Poland; g.gromadzka@uksw.edu.pl; 3 Department of Cellular Signalling, Mossakowski Medical Research Institute, Polish Academy of Sciences, 02-106 Warsaw, Poland; gczapski@imdik.pan.pl

**Keywords:** alpha-synucleinopathies, alpha-synuclein, Parkinson’s disease, SARS-CoV-2, COVID-19, neurodegenerative diseases

## Abstract

Alpha-synuclein (α-syn) is a 140-amino-acid, intrinsically disordered, soluble protein that is abundantly present in the brain. It plays a crucial role in maintaining cellular structures and organelle functions, particularly in supporting synaptic plasticity and regulating neurotransmitter turnover. However, for reasons not yet fully understood, α-syn can lose its physiological role and begin to aggregate. This altered α-syn disrupts dopaminergic transmission and causes both presynaptic and postsynaptic dysfunction, ultimately leading to cell death. A group of neurodegenerative diseases known as α-synucleinopathies is characterized by the intracellular accumulation of α-syn deposits in specific neuronal and glial cells within certain brain regions. In addition to Parkinson’s disease (PD), these conditions include dementia with Lewy bodies (DLBs), multiple system atrophy (MSA), pure autonomic failure (PAF), and REM sleep behavior disorder (RBD). Given that these disorders are associated with α-syn-related neuroinflammation—and considering that SARS-CoV-2 infection has been shown to affect the nervous system, with COVID-19 patients experiencing neurological symptoms—it has been proposed that COVID-19 may contribute to neurodegeneration in PD and other α-synucleinopathies by promoting α-syn misfolding and aggregation. In this review, we focus on whether SARS-CoV-2 could act as an environmental trigger that facilitates the onset or progression of α-synucleinopathies. Specifically, we present new evidence on the potential role of SARS-CoV-2 in modulating α-syn function and discuss the causal relationship between SARS-CoV-2 infection and the development of parkinsonism-like symptoms.

## 1. Introduction

Alpha-synucleinopathies are a group of neurodegenerative disorders characterized by the abnormal aggregation, accumulation, and spreading of α-synuclein (α-syn) in the brain and the other parts of the nervous system, like the spinal cord and enteric nervous system (ENS), where α-syn pathology may originate [1,2,3]. Under normal conditions, α-syn regulates synaptic function and the release of neurotransmitters [4]. However, under pathological conditions, it is prone to aggregation. Shorter forms of α-syn, like oligomers and protofibrils, gradually transform into fibrils, and finally, they become filamentous inclusions known as Lewy bodies (LBs), predominantly deposited in the neurons but also in the glial cells [5,6,7,8]. α-Synucleinopathies vary from each other by the affected brain regions and clinical manifestations. In addition to the most well-known α-synucleinopathy, Parkinson’s disease (PD), this group also includes several other conditions, such as dementia with Lewy bodies (DLBs), multiple system atrophy (MSA), pure autonomic failure (PAF), and REM sleep behavior disorder (RBD) [1]. Most α-synucleinopathy cases are sporadic, and their onset has been linked to an interaction between genetic susceptibility and environmental factors, such as viral infections and inflammation [9]. PD and other α-synucleinopathies have been specifically linked to the pandemic-scale outbreaks of infectious diseases [10]. Clinical case reports indicate a rise in parkinsonism diagnoses following the 1918 Spanish flu pandemic [11]. Moreover, it was suggested that viruses can initiate and accelerate the aggregation of α-syn leading to aberrant proteostasis [12]. Severe Acute Respiratory Syndrome Coronavirus 2 (SARS-CoV-2) is a member of the Coronaviridae family, characterized by its spherical, enveloped structure and RNA genome. The outbreak started in December 2019 in Wuhan City, China [13,14]. By February 2020, the World Health Organization (WHO) named the disease Coronavirus Disease 2019 (COVID-19). As of 2019, six coronaviruses were known to infect humans. Four of these (229E, OC43, NL63, and HKU1) typically cause mild respiratory illnesses, similar to the common cold. However, the remaining two—Middle East Respiratory Syndrome Coronavirus (MERS-CoV) and SARS-CoV—are associated with severe respiratory conditions that can progress to life-threatening acute respiratory distress syndrome (ARDS) [15,16]. SARS-CoV-2 could infect nearly everyone in the population within a short period and cause severe clinical states requiring specialized care in at least 10% of infected individuals. According to data from the WHO, there have been 776,007,137 confirmed cases, 7,059,612 deaths, and 13,642,098,070 vaccine doses administered [17].

**Figure 1:**
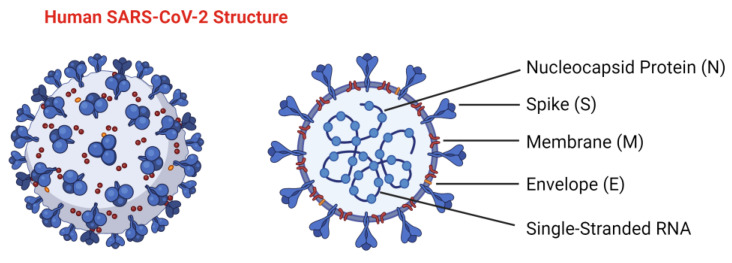
SARS-CoV-2 structure. Structural elements of the virus, including the spike (S) glycoprotein (homotrimer), membrane (M) protein, envelope (E) protein, and internal components, such as the viral single-stranded RNA and nucleocapsid (N) protein. Created in BioRender. B, L. (2024) BioRender.com/q94f155, accessed on 16 September 2024.

The SARS-CoV-2 genome encodes various proteins essential for the virus’s life cycle. These include non-structural proteins, such as 3-chymotrypsin-like protease, papain-like protease, helicase, and RNA-dependent RNA polymerase, all of which are key enzymes in the viral replication process. Additionally, it encodes several structural proteins. These include the spike (S) glycoprotein, composed of two subunits: S1 and S2. The S1 subunit contains the receptor-binding domain (RBD) responsible for attaching the virus to the host cell receptor, and angiotensin-converting enzyme 2 (ACE2), determining the virus’s host range. In contrast, the S2 subunit facilitates the fusion of the viral and host cell membranes. The membrane (M) protein is involved in nutrient transport across the viral membrane, and the envelope (E) protein plays a role in viral assembly and release. The nucleocapsid (N) protein not only packages the viral RNA but also interferes with the host’s innate immune response. Additionally, the virus encodes various accessory proteins that support its replication and pathogenicity [18,19] (Figure 1).

Since ACE2, which functions as the viral entry receptor, is expressed not only in the epithelial and endothelial cells of the lungs, heart, and intestines but also in various neuronal cell types, including dopaminergic (DA) neurons [20,21,22], the concerns regarding the potential neurological effects of COVID-19 have arisen. The incidence of COVID-19 is now increasing again. While the Omicron variant remains dominant, new subvariants, including Kraken, Pirola, JN1, KP, and the rapidly spreading KP.2, KP.3, and KP.1.1, collectively known as FLiRT (named after the first letters of the mutations in the specific proteins) have become an important burden [23,24].

**Figure 2:**
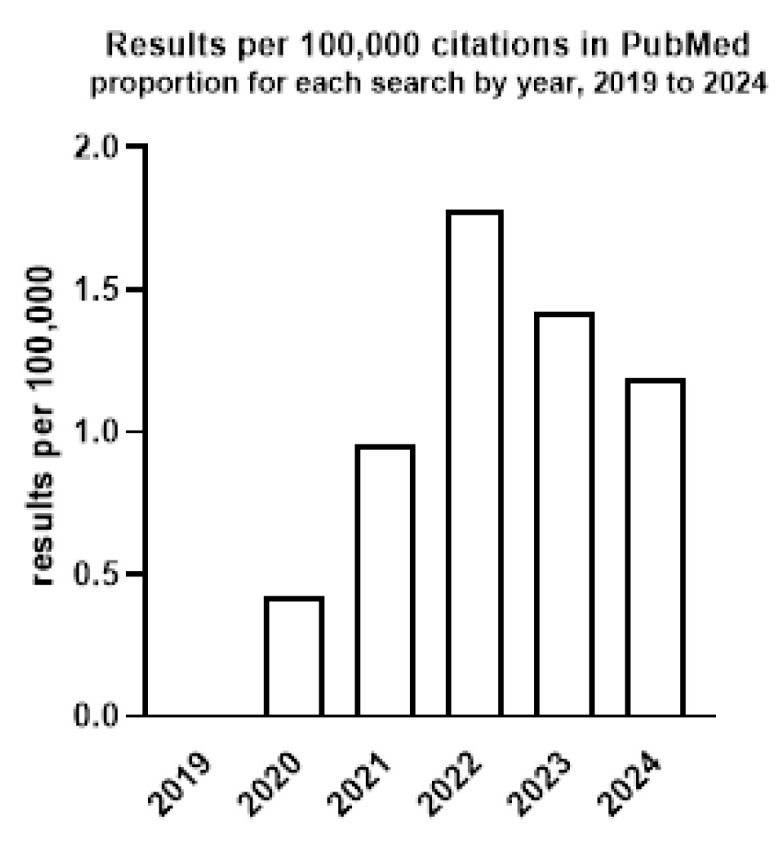
The number of published papers from 2019 to 2024 (until October 28.) containing the combined keywords “alpha-synuclein” and “COVID-19”. The results are presented as the proportion of citations per 100,000 in PubMed for each year. Data sourced using PubMed by Year: http://esperr.github.io/pubmed-by-year, accessed on 28 October 2024.

Individuals with pre-existing lung and heart conditions, as well as those over 60, are particularly vulnerable to severe complications [25]. Additionally, some COVID-19 patients experience neurological difficulties, such as olfactory disturbances and movement disorders, which are typical symptoms of PD [26]. Moreover, autopsy findings have shown damage to the density of DA neurons in the nigrostriatal pathway in some severe COVID-19 patients [20]. However, the relationship between SARS-CoV-2 infection and α-synucleinopathies is not well understood, and it is uncertain which disease is more prevalent than the other. Some evidence suggests that SARS-CoV-2 infection may be a potential risk factor for α-synucleinopathies, such as PD. Conversely, individuals with PD may be at risk for COVID-19 morbidity, as well as exposition to the severe course of the disease. The interest in the relationships between α-syn toxicity and COVID-19 infection in the context of the development of PD and other α-synucleinopathies has been increasing in recent years, as evidenced by the growing number of publications from 2019 to 2024 (Figure 2). Here, we discuss the current understanding of the relationship between SARS-CoV-2 infection and α-synucleinopathies, including PD.

## 2. The Relationship Between COVID-19 and PD

**Figure 3:**
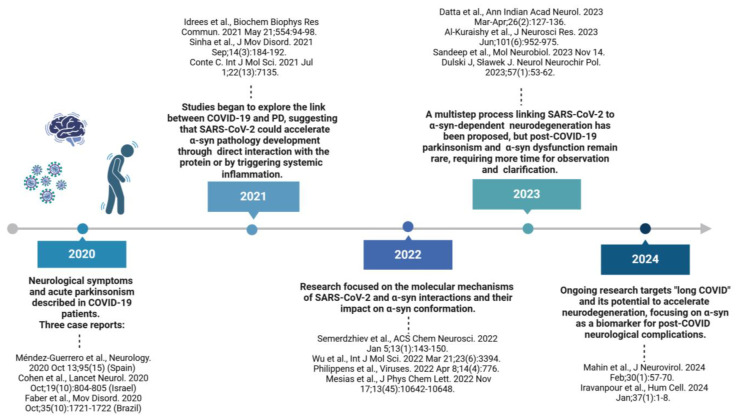
Timeline summarizing the research history on the relationship between α-synuclein and COVID-19 infection, highlighting key findings, significant years, and influential authors. References: 2020: [36,37,38]; 2021: [39,40,41]; 2022 [42,43,44,45]; 2023 [46,47,48,49]; 2024 [50,51]. Created in BioRender. Pan, I. (2024) https://BioRender.com/r11e475, accessed on 30 October 2024.

PD is the second most common neurodegenerative disorder, following Alzheimer’s disease (AD), with a rapidly increasing morbidity rate [27]. Currently, more than 10 million people worldwide are living with PD, but its prevalence is expected to increase to 12–17 million people by 2040 [28]. The presence of cytoplasmic LBs in surviving neurons and dystrophic Lewy neurites, along with the significant loss of DA neurons in the substantia nigra pars compacta (SNpc) and the reduced DA levels in the striatum, represents the primary pathological hallmark of PD [29]. The progression of PD is described by Braak et al. (2003) in six stages of α-syn pathology, which correlate with the development of both motor and non-motor symptoms [30]. The prodromal symptoms may originate from the degeneration of the vagus nerve’s dorsal motor nucleus, the olfactory system, and the ENS, including Meissner’s and Auerbach’s plexuses, where α-syn pathology is first detected [31]. Pathology originating within the ENS is now recognized as a crucial component of the gut–brain axis in PD pathogenesis [32]. Consequently, the clinical presentation of PD includes motor symptoms, such as bradykinesia, muscle rigidity, postural instability, and resting tremor, as well as pre-motor and non-motor symptoms. Pre-motor symptoms like constipation, insomnia, and depression can precede the clinical diagnosis by up to 20 years. Non-motor symptoms, which include neuropsychiatric, cognitive, autonomic, sleep-related, and sensory abnormalities, may manifest from the prodromal phase through to the advanced stages of PD [33,34]. Viral infections are widely recognized as a potential risk factor for idiopathic PD and secondary parkinsonism, suggesting a possible shared mechanism for both PD and COVID-19 [35]. Age and underlying health conditions constitute further risk factors, which could make PD patients more vulnerable to harsh outcomes in case of infection with SARS-CoV-2. Simultaneously, inflammation and stress caused by a severe infection might cause worse PD symptoms. Research since 2020 has gradually shed light on a potential relationship between α-syn pathology and COVID-19 infection. The evolving findings suggest that COVID-19 may exacerbate neurodegenerative processes by promoting neuroinflammation, which in turn leads to α-syn oligomerization and aggregation. A direct interaction between SARS-CoV-2 proteins and α-syn has also been proposed to accelerate its aggregation. Ongoing research aims to further elucidate these links and better understand the long-term neurological risks associated with COVID-19 (Figure 3).

### 2.1. Parkinsonism as a Consequense of COVID-19

While the number of studies reporting a link between parkinsonism and COVID-19 infection is increasing (Table 1), it is too early to conclude that SARS-CoV-2 directly induces PD [52,53,54]. The occurrence of parkinsonism during or immediately after SARS-CoV-2 infection is a rare clinical complication, and the most likely scenario involves a multifactorial “umbrella syndrome” [48,55].

However, the meta-analysis of serum/plasma mass spectrometry-based proteomics data from 15 datasets with a total of 538 COVID-19 patients and 523 healthy controls presented the proteomic profiling evidence linking COVID-19 to neurological complications, particularly AD and PD. The authors identified a direct correlation in the expression patterns of 23 proteins engaged in PD with COVID-19. Importantly, a further protein–protein interaction network and cluster analysis identified α-syn as a hub protein [50].

On the other hand, the studies analyzing the impact of COVID-19 on α-syn serum/CSF levels in patients with neurological symptoms deny the theory of α-syn upregulation in humans with PD and other neurological symptoms following SARS-CoV-2 infection [56]. The authors obtained no significant changes in serum total α-syn level between three analyzed groups, i.e., COVID-19 patients with generalized myoclonus (encephalopathy or parkinsonism), related to SARS-CoV-2 infection, but not with the other diseases; age/sex-matched COVID-19 patients, but free of even milder neurological complaints such as anosmia or headache, who were admitted to the hospital in the same period; and age/sex-matched healthy controls. CSF α-syn level was also comparable between the neurological COVID-19 patients and the healthy individuals.

**Table 1:** The impact of COVID-19 on the occurrence of PD.

Aim	Method/Subject of Analysis	Results	Reference
Meta-analysis of serum/plasma proteomic data from COVID-19 patients assaying the links between SARS-CoV-2 infection and neurological disorders, specifically AD and PD.	Mass spectrometry-based proteomics data with a total of 538 COVID-19 patients and 523 healthy controls.	Analysis confirmed a direct correlation in the expression patterns of 24 proteins implicated in AD and 23 proteins involved in PD with COVID-19. A protein–protein interaction network and cluster analysis revealed direct correlation in differential expression between COVID-19 and PD and identified SNCA as a hub protein.	[50]
Meta-analysis answering the question: Are the new-onset neurodegenerative diseases long-term sequelae of the SARS-CoV-2 infection?	Articles published up to 10 January 2023. Twelve studies involving 33,146,809 individuals (2,688,417 post-COVID-19 cases and 30,458,392 controls).	The significant correlation between SARS-CoV-2 infection and increased risk for new-onset AD (Hazard Ratio [HR] = 1.50), dementia (HR = 1.66), and PD (HR = 1.44).	[57]
The systematic review concentrated on the impact of post-SARS-CoV-2 immune-mediated responses/the host’s altered immune counter-offensive on the occurrence of neurodegenerative diseases like PD in a complex interrelation between genetic and epigenetic risk factors.	A synthetic and systematic literature review based on the “Preferred Reporting Items for Systematic Principles Reviews and Meta-Analyses” (PRISMA) methodology; 104 papers were finally selected.	It is too early to establish if the neuroinflammatory events accompanied by COVID-19 could activate long-term neurodegenerative consequences and lead to new cases of PD occurrence and the worsening of the existing disease outcome. Further clinical and prospective longitudinal cohort studies are required.	[58]
The analysis of the possible mechanisms involved in COVID-19-induced neuropathology like PD. The analysis of pathways involved in the downregulation of ACE2 following SARS-CoV-2 infection and its effect on PD progression.	The analysis of the pathways involved in the downregulation of ACE2 following SARS-CoV-2 infection and its effect on PD progression. The molecules and chemicals associated with COVID-19 and PD were subjected to Ingenuity Pathway Analysis (IPA) “Grow”; 81 overlapping molecules between COVID-19 and PD were further subjected to IPA’s “Core Analysis” tool to identify the upstream regulators and signaling pathways.	Core Analysis revealed the neuroinflammation signaling pathway (NISP) to be one of the principal signaling pathways involved and SNCA as the top upstream controller associated with both COVID-19 and PD. A network connectivity pathway map of the downstream effects of COVID-19 revealed that ACE2 blocking upregulates SNCA expression, potentially accelerating PD progression.	[59]
A systematic review and meta-analysis of studies reporting parkinsonism cases among patients recovering from COVID-19.	Research from seven major databases covering a timeline of 1 January 2020 to 1 January 2022. Ten studies met the inclusion criteria and covered thirteen patients with a median age of 60.0. There were eight males (61.5% of patients), and 53.8% of individuals were documented to have at least one comorbidity. Fisher’s exact test was used to examine the factors connected with COVID-19 and parkinsonism as its results.	Indication of parkinsonism as post-COVID-19 neurological sequelae. Cogwheel rigidity was the most common manifestation of parkinsonism in eleven patients. The most standard medicine modality used was Levodopa (76.9% of cases). Ten patients (76.9%) with bradykinesia achieved a complete recovery.	[60]

What we know for sure is that the neurological complications following viral infections are prevalent [61]. In recent years, there has been frequent documentation of new or worsening non-motor symptoms in PD patients infected with SARS-CoV-2. Some of these symptoms, such as depression, anxiety, cognitive impairment, pain, and fatigue, have also been identified as components of Post-COVID-19 Syndrome (PCS), commonly known as Long-COVID-19 [62]. However, distinguishing between PCS in PD patients and a simple exacerbation of PD symptoms secondary to COVID-19 is challenging. Bougea et al. (2023) found a significant worsening of motor function, as measured by the Unified Parkinson’s Disease Rating Scale Part III (UPDRS III), both at baseline and six months after COVID-19 infection in PD patients with PCS symptoms. The most common manifestations in these patients were anosmia/hyposmia and a sore throat, followed by dysgeusia and skin rashes. However, no significant differences were found in motor and non-motor clinical measures or the levodopa equivalent daily dose (LEDD) between PD patients with and without PCS, indicating that no prognostic factor for PCS in PD patients has been identified [63]. However, another study suggested that fatigue and the duration of the infection were linked to the exacerbation of motor and non-motor symptoms in PD patients during the Omicron pandemic [64].

The decline in mental health and exacerbating symptoms like anxiety and depression among people with PD due to COVID-19 restrictions may be related to difficulties in accessing regular healthcare, the loss of systematic physical activity, and social isolation [65,66]. Interestingly, another report indicated that apathy and anhedonia in PD patients may have been mitigated by increased social support from families isolated at home during the COVID-19 pandemic [67]. In summary, the connections between PD and COVID-19 prevalence are being extensively studied, but there is still no evidence that COVID-19 directly causes PD. The area of the interrelated neuropathogenetic phenomena remains complicated and multifaceted, requiring further investigation.

### 2.2. The Prevalence, Outcomes, and Prognosis of COVID-19 in Patients Diagnosed with PD

In the last four years, numerous systematic reviews and meta-analyses have been published regarding the outcomes of COVID-19 among PD patients (Table 2). In summary, several meta-analyses indicated that patients with neurodegenerative diseases and mild cognitive impairment represent a group at a high risk of acquiring SARS-CoV-2 and may be disproportionately affected by severe outcomes of COVID-19 once infected [68]. However, the majority of statistical inference results did not provide evidence that PD predisposes individuals to poor COVID-19 outcomes [69]. Although hospitalization and mortality rates are relatively high among PD patients in some meta-analyses, these may be attributed to advanced age and numerous comorbidities in patients diagnosed with PD [68].

### 2.3. The Theory of SARS-CoV-2 Neuroinvasion in PD

**Figure 4:**
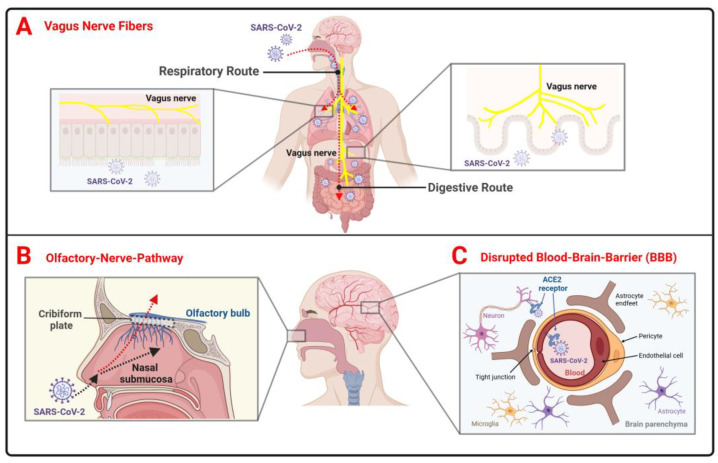
Key neuroinvasive pathways that SARS-CoV-2 may use to reach the CNS. SARS-CoV-2 may potentially gain access to the brain through three primary routes. (**A**) **Vagus nerve fibers**: SARS-CoV-2 can be transmitted via the respiratory system through saliva droplets or nasal discharge toward the lungs, as well as through the gastrointestinal tract via swallowed saliva or the consumption of contaminated food, where the virus is known to replicate. ACE2 expression is high in both the lungs and gastrointestinal epithelium, making these tracts vulnerable to SARS-CoV-2 entry. The vagus nerve extends from the brainstem to various organs, including the lungs and digestive tract. Consequently, the virus may travel retrogradely along nerve fibers from the respiratory and gastric epithelium via the vagus nerve to the brainstem. (**B**) **Olfactory nerve pathway**: SARS-CoV-2 can enter the nasal cavity, travel into the nasal submucosa, and infect olfactory sensory neurons in the nasal epithelium. The virus may then travel along olfactory nerve fibers, reaching the olfactory bulb by moving upstream. This pathway could explain symptoms such as anosmia and may be a key route for the virus to spread to other parts of the CNS. (**C**) **Disrupted blood–brain barrier (BBB)**: SARS-CoV-2 infection can trigger a strong immune response, leading to inflammation that may increase the permeability of the blood–brain barrier, facilitating the virus’s entry into the CNS. ACE2, expressed on endothelial cells, pericytes, and some neurons in the brain, could mediate viral entry into the brain. Created in BioRender. Pan, I. (2024) https://BioRender.com/r11e475, accessed on 30 October 2024.

The hypothesis of neuroinvasion by SARS-CoV-2 arises from the detection of virus RNA in the cerebrospinal fluid (CSF) of COVID-19 patients [78]. Although research into how the virus may invade the nervous system is ongoing, several key neuroinvasive pathways have been proposed. The virus might enter the central nervous system (CNS) directly through the olfactory nerve pathway, using the vagus nerve through its terminals located along the respiratory and gastrointestinal tract, as well as via the neuroinflammation-affected blood–brain barrier (BBB) [21,79] (Figure 4).

It is worth mentioning that olfactory dysfunction is a highly acknowledged feature of both PD and COVID-19 [80]. The olfactory epithelium (OE) consists of a heterogeneous cell population, including the olfactory sensory neurons, basal, supporting cells (SUS), and microvillar and glandular cells. One or several of these cell types could serve as a potential point of SARS-CoV-2 entry into the OE and thus determine the underlying chain of events leading to anosmia. High expression of ACE2 in the OE facilitates the entry of SARS-CoV-2 into the central nervous system [81]. ACE2 is highly expressed in SUS cells, Bowman’s gland, and microvillar cells [82,83,84,85,86]. Moreover, protease TMPRSS2, which is a well-established host factor in facilitating SARS-CoV-2 entry upon binding to ACE2, has also been reported to be expressed in the SUS cells [87,88].

In addition to being one of the diagnostic symptoms for prodromal PD, the degree of olfactory dysfunction can also be a good marker of its progression [89]. Similarly, a sudden decline or complete loss of olfactory sensitivity is now considered a common early symptom of COVID-19, while parosmia and phantosmia are increasingly recognized as a Long-COVID-19 symptoms [90]. α-Syn may be an important player in olfactory impairment. The overactivated microglia responses in the olfactory bulb, followed by the accumulation of hyperphosphorylated α-syn and tau protein in the cortex, were reported in the intranasally SARS-CoV-2 infected hamster model [91]. Importantly, later studies indicated that microglia cell density and immunoreactivity of α-syn in female hamsters appeared biphasic, with an initial decline at six days post-infection followed by microgliosis and α-syn accumulation at three weeks post-infection. The most pronounced changes were reported in the amygdala and striatum, regions affected early in PD [92].

The SARS-CoV-2 virus has been detected in the brains, including the SN of COVID-19 patients during autopsies [20,93]. These neuronal populations are extremely vulnerable to SARS-CoV-2 neuroinvasion, partly because DA neurons express ACE2 receptors, which may facilitate viral entry [20,21,22]. The ACE2 receptor is located on neuronal and glial cells of several brain structures, including the SN and striatum [94]. For example, within the rat brain, double ACE2- and tyrosine hydroxylase (TH)-positive cells were found in the SNpc and ventral tegmental area (VTA) regions [22]. TH is the rate-limiting enzyme for DA synthesis and the marker of DA cells. Furthermore, the co-expression between ACE2 and DOPA decarboxylase, another enzyme, implicated in DA production was described. As ACE2 expression is downregulated by the SARS-CoV infection, the virus may affect DA synthesis [21]. It is important to note that SARS-CoV-2 may use DA receptors to enhance its life cycle, raising viral entry opportunities. Furthermore, the virus, via DA-mediated disturbance of intracellular biosynthesis, may restrict the innate and adaptive immune responses [95].

Furthermore, DA neurons in the SNpc are especially prone to oxidative damage due to their distinct characteristics: DA metabolism, high levels of iron and neuromelanin, and a reduced antioxidant capacity evidenced by lower levels of the key endogenous antioxidant, reduced glutathione, in this brain region [96]. Oxidative stress in these neurons is further exacerbated by mitochondrial dysfunction, which accumulates with aging [97]. Additionally, nigrostriatal DA neurons have high cellular energy demands to maintain elevated basal oxidative phosphorylation in the mitochondria, along with high axon terminal density and extensive axonal arborization. Furthermore, the SNpc contains the highest density of microglia among all brain regions—up to ten times denser in humans—making nearby neurons more vulnerable to uncontrolled microglial activation and subsequent neurodegeneration [98]. A senescence-like phenotype, characterized by markers such as lipofuscin accumulation, changes in senescence-associated genes, increased lysosome numbers, mitochondrial dysfunction, and elevated protein oxidation has been identified in SARS-CoV-2-infected human pluripotent stem cell-derived DA neurons [20]. Furthermore, a reduction in TH- and neuromelanin-positive DA cell bodies and nerve fibers were observed in severe COVID-19 patients, highlighting the sensitivity of this type of cell to SARS-CoV-2 infection [20]. Given the above-mentioned specific properties of the DA neurons, the cellular stress induced by COVID-19 infection may push these vulnerable cells past the threshold of neurodegeneration. However, the molecular mechanisms underlying COVID-19-induced neuronal cell death remain complex. The interplay between heightened neuroinflammation, oxidative stress, and mitochondrial dysfunction is closely associated with α-synucleinopathies, as well as with COVID-19 pathomechanism. This creates a vicious cycle, leading to progressive neuropathological manifestations. Moreover, these harmful processes are also key drivers in promoting α-syn oligomerization, aggregation, and toxicity.

### 2.4. The Role of SARS-CoV-2 in α-Syn Alterations

**Figure 5:**
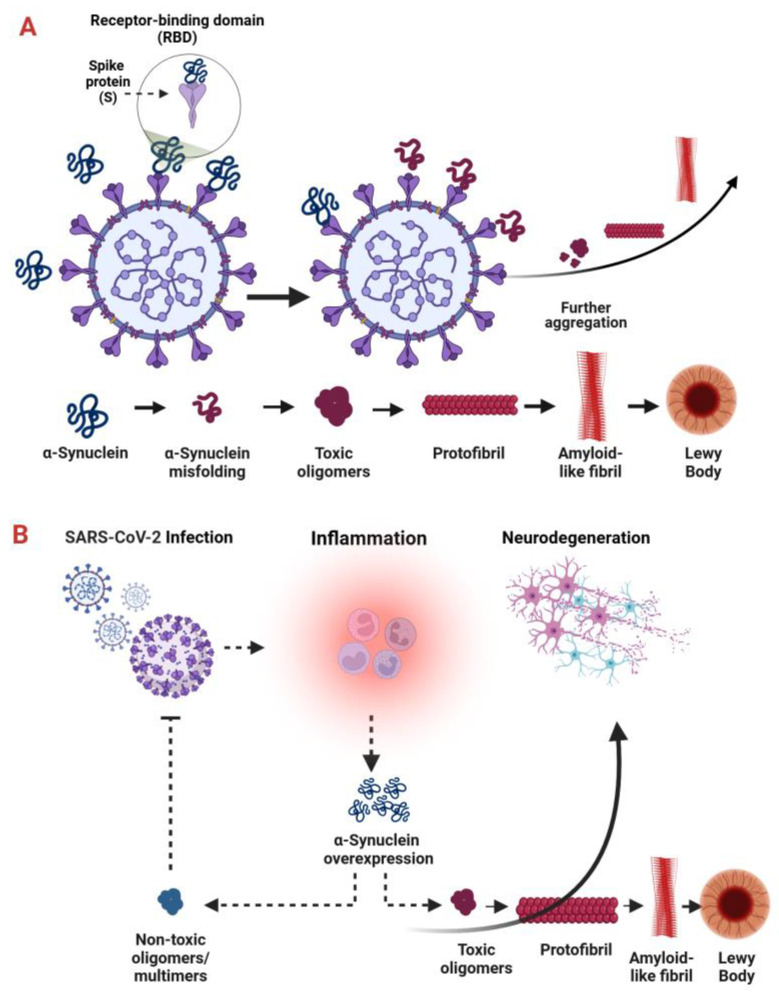
SARS-CoV-2–induced alterations in α-synuclein. (**A**) Potential direct interactions between the SARS-CoV-2 spike (S) protein and α-synuclein. The S protein may directly interact with α-synuclein, potentially altering its structure and increasing its propensity to misfold and aggregate. This interaction could encourage the formation of toxic α-synuclein oligomers or fibrils, which are harmful to neurons. (**B**) SARS-CoV-2–induced activation of α-synuclein’s immunomodulatory function. α-Synuclein plays a role in the immune system, including the modulation of inflammatory responses. When SARS-CoV-2 infects a cell, it initiates inflammatory signaling that involves microglial activation and the release of high levels of pro-inflammatory cytokines and chemokines. This inflammatory response may lead to α-synuclein accumulation and neuronal damage. Conversely, non-toxic multimers of α-synuclein could help prevent viral spread. Created in BioRender. Pan, I. (2024) https://BioRender.com/r11e475, accessed on 30 October 2024.

In light of the relationship between viral infections and the development of neurodegenerative processes, several studies have focused on the role of virus-induced alterations in α-syn oligomerization and aggregation. Infections evoked by viruses such as the human immunodeficiency virus (HIV), West Nile Virus (WNV), Venezuelan equine encephalitis virus (VEEV), Influenza (H1N1), and SARS-CoV-2 can trigger α-synucleinopathies in the CNS [99,100]. Therefore, α-syn has been proposed as a key factor linking the pathomechanisms of PD and COVID-19 [47,51]. Although more in vivo studies are showing that SARS-CoV-2-related changes in α-syn mimic the neuropathological features seen in PD patients [92,101], the specific ways in which SARS-CoV-2 affects α-syn regulation and the effects of these interactions are still not well understood. However, two main pathways have been proposed for how SARS-CoV-2 might alter α-synuclein: (1) direct interaction between the virus and α-synuclein, and (2) activation of the immunomodulatory function of α-syn (Figure 5).

A direct molecular interaction between SARS-CoV-2 proteins and α-syn, which could initiate a conformational shift in the soluble monomeric protein and accelerate the formation of toxic multimeric species, including oligomers/protofibrils and fibril aggregates, is likely due to the presence of specific viral proteins, such as the S and N proteins, which may interact with α-syn [43,101,102]. The RBD of the SARS-CoV-2 S1 contains multiple heparin-binding sites that interact with heparin and heparin-binding proteins (HBPs), including proteins prone to aggregation. It is therefore suggested that α-syn could recognize these heparin-binding sites on the S protein, bind to them, and subsequently undergo oligomerization, aggregation, and fibrillation. The ability of the S1 protein to drive α-syn aggregation has been confirmed both in in vitro and in rodent models, with neuroinflammation emerging as a necessary trigger [101]. A signal from the ionized calcium-binding adapter molecule 1 (Iba-1), being an activated microglia marker, correlated with the α-syn aggregation in the SNpc region of S1-administered rat brains. Similarly, S1-conditioned media from BV2 microglia cells increased both the α-syn monomer level, its phosphorylation on Ser129, and aggregated the form burden via a pro-inflammatory mediated mechanism. It is important to note that about 90% of the accumulated α-syn in LBs is phosphorylated at Ser129 [103]. In addition, S1 can directly accelerate α-syn aggregation through increasing mitochondrial reactive oxygen species (ROS) under conditions of its sufficient accumulation. Finally, S1 exerts a synergistic effect with DA neurotoxin 1-methyl-4-phenylpyridinium (MPP+), which is accompanied by excessive ROS production, mitochondria damage, and reduced viability of the DA neurons [101]. Bioinformatic analysis of the protein–protein interactions revealed that the α-syn–S1 complex exhibited the strongest binding affinity, followed by the α-syn–N protein complexes. It was observed that infection with SARS-CoV-2 S and N proteins led to Lewy-like pathology in vitro, as evidenced by increased α-syn phosphorylation and aggregation in HEK293 cells overexpressing α-syn, particularly in N protein co-transfected once [43]. Another study demonstrated that the SARS-CoV-2 N protein catalyzed the formation of α-syn fibrils in a two-step aggregation process. Initially, the mixed population of α-syn/N-protein fibrils arose, which in the case of the N protein lack transformed into pure α-syn fibrils. In the second phase, fibrils continue their growth until a new balance was acquired [102].

Virus-induced α-syn oligomerization, aggregation, and subsequent accumulation—observed for SARS-CoV-2 and other viruses such as H1N1 and HIV—could accelerate neuroinflammation and heighten the risk of developing neurodegenerative processes. This aligns with the potentially toxic effects of altered α-syn in neurons [12,44,104]. Fibrillar α-syn was demonstrated to activate microglial nucleotide-binding leucine-rich repeat receptors (NLRs) family pyrin domain containing 3 (NLRP3) inflammasome [105]. Further studies identified that NLRP3 can be activated also by SARS-CoV-2 S-glycoprotein in Lipopolysaccharide (LPS)-primed microglia in a mechanism dependent on an ACE2 and nuclear factor kappa B (NF-κB). Furthermore, the presence of α-syn fibrils was found to enhance S protein-mediated microglial inflammasome activation, which was completely abolished by NLRP3-inhibition [56]. These findings confirm the potential synergistic effect of the SARS-CoV-2 S protein and α-syn. Consequently, NLRP3 inhibitors, which are currently in clinical development for neurodegenerative diseases including PD, could be considered for the treatment of SARS-CoV-2-induced neurological manifestations [105].

SARS-CoV-2 infection may also impact α-syn clearance. Several viral proteins can induce endoplasmic reticulum (ER) stress and activate unfolded protein responses (UPRs) to maintain cellular homeostasis. It has been suggested that SARS-CoV-2 may impair proteostasis through a mechanism dependent on the open reading frame 8 (orf8) protein [106]. It may lead to dysregulated ER protein trafficking and hence to uncontrolled α-syn burden and aggregation [107].

As previously mentioned, although α-syn accumulation contributes to cell toxicity and the development of neuropathological manifestations, it also plays a crucial role in sustaining antiviral innate immunity. The upregulation of α-syn in neurons is a normal response to RNA virus infections and is an essential component of the host’s immune response [108]. The immunomodulatory properties of α-syn were evidenced by its increased neuronal expression following acute infection with WNV or VEEV [51,107]. α-Syn localized to the ER-derived membranes regulates ER-induced stress, restricts viral replication, and thus protects CNS [109]. Moreover, in α-syn-knockout mice inoculated with WNV and VEEV strain TC-83, the infectious titer in the brain was increased compared with the wild-type and heterozygote littermates. Moreover, the mortality of α-syn-knockout mice was also increased compared with the control animals [109]. In a rodent model of the peripheral H5N1 influenza infection, persistent CNS microglial activation and abnormal α-syn phosphorylation that lasts long after infection resolution were observed. Simultaneously, the loss of DA neurons in the SNpc was observed 60 days after infection [110]. It is probable that, in response to SARS-CoV-2 infection, α-syn upregulation may also occur, which may be explained as a protective mechanism against virus replication. Using α-syn knock-out mice and human neuronal models, α-syn was shown to be a neuron-specific modulator necessary to complete interferon-stimulated gene expression in neurons after acute RNA virus infection [111]. Moreover, virus infections increased the level of α-syn, phosphorylated on Ser129 in human and non-human primate neuronal tissues, linking responses to virus infection with accumulation of phosphorylated α-syn [111].

α-Syn is expressed predominantly within CNS, but its role in the peripheral tissues is also important in the context of antiviral defense. It is worth highlighting that α-syn is the key player in peripheral cells’ response to viral infection, which may prevent virus transmission from the peripheral nervous systems (PNSs) to the CNS and slow down neuroinvasion. The data of Limanaqi et al. (2024) indicated that α-syn upregulation in lung epithelial cells occurs as a type-I interferon (IFN)-related response to SARS-CoV-2 infection, which participates in the suppression of viral replication. The dynamic of α-syn conformation/aggregation, leading to the production of a non-toxic multimer, is crucial in IFN-related responses. Extracellular α-syn monomers accelerated SARS-CoV-2 replication and reduced both IFN-related genes and the α-syn multimer/monomer ratio. On the other hand, IFN-β treatment restricted virus replication and increased α-syn multimers levels in the lack of cell toxicity. Moreover, in endothelial cells expressing abortive SARS-CoV-2 replication, α-syn multimerization was not altered following exposure to the virus, suggesting that only productive viral infection impairs α-syn aggregation [112].

However, the hypothesis of α-syn accumulation as a protective antiviral factor has simple limitations. The initial upregulation of this protein may be a trigger for its further oligomerization, seeding, and subsequent propagation, ultimately resulting in widespread neurodegeneration. Therefore, α-syn may have a dual role following infection by various viruses, including SARS-CoV-2, with the type of α-syn conformers and their dynamics being key factors in modulating the course of infection (Figure 5).

### 2.5. Promising Therapeutic Targets for Both PD and COVID-19

Since PD and COVID-19 share some molecular and cellular pathological mechanisms, particularly those related to inflammation, oxidative stress, mitochondrial dysfunction, and immune response, several therapeutic targets and strategies have been proposed to modulate these pathways in both diseases. The most promising approaches focus on reducing inflammation—such as using inhibitors of the NLRP3 inflammasome, IL-6, or TNF-α, which are being tested for their ability to dampen the excessive immune response in both conditions—and addressing oxidative stress and mitochondrial dysfunction [113,114,115]. Additionally, enhancing autophagy with drugs like rapamycin or metformin could provide therapeutic benefits for both PD and COVID-19, as could targeting the microbiome by modulating it with probiotics or other gut-targeted therapies [116,117,118].

Recent data suggest that antagonists of purinergic receptors may serve as potential treatments for reducing inflammation and oxidative stress in both PD and COVID-19. The P2X purinoceptor 7 (P2X7R), a crucial mediator of neuroinflammation, is particularly promising. The study of Hasan et al. (2022) demonstrated that readily available P2X7R antagonist lidocaine can be beneficial in restoring regular immune function during severe COVID-19 infection [119]. To overcome the trouble of the EC50 for P2X7R inhibition of lidocaine significantly exceeding the maximal tolerable plasma concentration where adverse effects begin, the authors selectively inhibited the P2X7Rs of the immune cells of the lymphatic system generating a clonal expansion of Tregs in the local lymph nodes. Tregs migrating throughout the organism ameliorated hyperinflammation, which was described in six critically suffering COVID-19 patients. Blocking the P2X7R may help to alleviate the uncontrolled ATP release to the extracellular space, cytokine storm triggered by the P2X7R/NLRP3 inflammasome axis, and lysosomal dysfunction. Moreover, reduced levels of ATP and the Ca^2+^ influx, regulated by P2X7R, lessened the possibility of α-syn aggregation [120]. It is important to note that another potential target in PD is the P2X1 receptor (P2X1R), which is highly expressed in the SN and striatum, regions affected by PD. P2X1R is involved in α-syn accumulation, a process linked to lysosomal pH elevation and dysfunction caused by high levels of extracellular ATP [121].

When discussing potential common treatments for PD and COVID-19, amantadine cannot be overlooked. Primarily used in the management of PD, amantadine increases extracellular DA levels by inhibiting its reuptake by presynaptic neurons and antagonizing the N-methyl-D-aspartate (NMDA) receptor function [122]. Its pharmacological activities are unique in incorporating dopaminergic and antiglutamatergic effects, which direct its dual action on parkinsonian-like symptoms and levodopa-induced dyskinesias [123]. Currently, amantadine, including its long-acting formulations, is mainly employed as an add-on therapy to mitigate levodopa-induced dyskinesias [124,125]. Amantadine was also one of the first antiviral drugs approved for the treatment of Influenza A, though its use has been limited in many countries due to the emergence of resistant viral strains [126]. While SARS-CoV-2 infection is not an official indication for amantadine, numerous studies highlight its potential, especially as a candidate for neurological manifestations management in patients with coexisting parkinsonism and other neurological disorders [127,128,129]. Protein E (envelope) representing one of the SARS-CoV viroporins involved among others in the NLRP3 inflammasome activation may be an important target for antiviral therapy [130]. Amantadine was shown to inhibit the ion channel activity of SARS-CoV-2 Protein E [131]. Another in vitro study demonstrated the effectiveness of amantadine in inhibiting the replication of SARS-CoV-2. Still, the IC50 value exceeded the therapeutic concentration of amantadine after systemic administration but did not exclude the inhalation or intranasal management route [132]. The potential role of amantadine in treating COVID-19 remains a topic of public debate. However, results from a retrospective, multicenter cohort study among patients with idiopathic PD, comparing those who used amantadine chronically to those who did not, indicated that amantadine did not influence the incidence or severity of COVID-19 [133]. Similarly, a randomized, double-blind, placebo-controlled, single-center clinical trial found no effect of amantadine on the progression of the disease following SARS-CoV-2 infection [134]. On the other hand, a hospital-based, observational, retrospective cohort study reported the significantly lowered impact of amantadine pre-exposure use among PD and Multiple Sclerosis (MS) patients on COVID-19 infections [135]. Moreover, a clinical trial evaluating the effect of amantadine on post-COVID-19 fatigue showed a statistically significant decline in fatigue levels, suggesting a potential beneficial effect of the drug [136]. Similarly to the promising effects of amantadine on fatigue, memantine, another well-known adamantane derivative used in AD therapy, may have a beneficial impact on cognitive impairment, another frequent COVID-19 sequela [137].

Calcium homeostasis is also regulated by lithium. Its chronic treatment was confirmed to significantly lower intracellular calcium flux, specifically by activating metabotropic glutamatergic receptor 5 (mGluR5) [138]. Lithium, being a mood stabilizer and the first-choice treatment option for bipolar disorders, may be a valid neuroprotective therapeutic also in neurodegenerative diseases like PD and AD, which was indicated in a meta-analysis of pre-clinical and clinical studies [139]. Moreover, the antiviral effects of lithium were investigated using electronic health records of 14,008 individuals with a documented therapeutic lithium level (mean level 0.741 mmol/L) and 12,546 individuals with a recorded subtherapeutic lithium level (mean level 0.352 mmol/L) during the COVID-19 pandemic. It was demonstrated that the 6-month COVID-19 infection incidence was lower among matched patients with therapeutic versus subtherapeutic lithium levels, indicating the association between higher lithium levels and a lower risk of COVID-19 [140].

Neuroinflammation is an extremely important background of both COVID-19 and PD. Therefore, reducing the hyperstimulated microglia activity by naltrexone, unique for its low doses, is another vital target for both diseases. Naltrexone is a Food and Drug Administration (FDA)-approved non-peptide opioid receptor antagonist, which in standard doses is used to treat symptoms of alcohol and opioid abuse. However, the finding of a non-neuropsychiatric component of opioid receptor signaling with low-dose naltrexone (LDN) has discovered its novel applications in inflammation-associated conditions treatments [141]. The mechanism of LDN action probably via Toll-like receptor 4 (TLR-4) antagonism widely expressed on microglial cells relies on the switch to the anti-inflammatory and neuroprotective M2-phenotype [142]. LDN was reported to reduce both LPS-induced cytokine storm and extracellular signal-regulated kinase (ERK 1/2) phosphorylation/activity. ERK1 and ERK2 are mitogen-activated protein kinases (MAPKs) regulating cell proliferation and differentiation in response to extracellular stimuli, like cytokines, which have been shown to activate virus replication. Moreover, virtual docking simulation data have shown that LDN is able to disrupt the SARS-CoV-2-RBD-ACE2 complex, playing a critical role in virus invasion and virulence [143].

Another therapeutic target for both PD- and COVID-19-related neurological complications is ferroptosis inhibition. Ferroptosis is an iron-dependent programed cell death, which when overactivated leads to oxidative stress and uncontrolled inflammation. A lower level of glutathione, along with the deregulation of glutathione peroxidase 4 (GPX4) and the accumulation of lipid peroxides, is a critical mechanism of ferroptosis [144]. As mentioned above, the SNpc contains a high concentration of iron and, compared with other structures, a relatively low level of reduced glutathione, which serves as an antioxidant defense [96]. Thus, the markers of ferroptosis highly overlap with the neuropathological features of PD. Iron is a required co-factor for multiple essential enzymes and is necessary for viruses’ efficient replication. Cellular iron burden synergistically increased reactive oxygen and nitrogen species, pro-inflammatory cytokines and chemokines production in cells primed with the S protein [145]. Simultaneously, iron and ferritin overload coexisting with inflammation during SARS-CoV-2 infection were detected in severe types of COVID-19 [146]. Therefore, ferroptosis is also suggested to be a vital mechanism of cell death in COVID-19-related parkinsonism [21]. Cell death, including ferroptosis, is generally beneficial because it can inhibit virus multiplication. However, uncontrolled oxidative stress and inflammation lead to multi-organ dysfunction, including BBB breakdown, brain injury, and neurological symptoms. The major treatment strategy to restrict ferroptosis is by preventing the Fenton reaction and oxidative damage like lipid peroxidation by managing iron chelators such as deferoxamine [146,147,148] and lipophilic antioxidants like ferrostatin-1 and liproxstatin-1 [149]. Deferoxamine was reported to improve the clinical status and lessen the hospital mortality rate in patients with COVID-19 admitted to the intensive care unit [148]. The common therapeutic targets of PD and COVID-19 are summarized in Table 3. However, further research is needed to fully understand the connections between both disorders to develop effective treatments for coexisting conditions.

## 3. The Connection Between COVID-19 and α-Synucleinopathies Other Than PD

Although the connection between SARS-CoV-2 and α-syn is well documented, the relationship between COVID-19 and α-synucleinopathies other than PD remains speculative. Currently, there are only a limited number of reports linking this group of diseases with COVID-19. However, cases of COVID-19 patients diagnosed with certain α-synucleinopathies indirectly suggest a causal relationship between viral infection and α-syn pathology. The distinction between PD and other α-synucleinopathies, such as DLB and MSA, is based primarily on differences in symptoms or the timing of their onset. Nonetheless, all these diseases share a common pathomechanism, where neuroinflammation plays a critical role following the accumulation of α-syn [1]. Table 4 summarizes the case of patients diagnosed with α-synucleinopathies other than PD after COVID-19 infection.

### 3.1. DLB

DLB is recognized as the second most common cause of dementia, after AD. Clinically, DLB is distinguished from PD dementia primarily by the timing of the onset of dementia relative to parkinsonism [159]. In DLB, cognitive impairment appears early, either preceding parkinsonian motor signs or developing within one year of their onset, while PD dementia (PDD) is diagnosed when dementia emerges in well-established PD. Most DLB patients initially exhibit frontal lobe dysexecutive syndrome, visual hallucinations, disorientation, and delusions, followed later by motor symptoms such as gait impairments, imbalance, and, less commonly, symmetrical tremors [160,161]. An exacerbated inflammatory response within the CNS is known to accelerate DLB development. In a non-human primate model, SARS-CoV-2 infection was shown to cause brain inflammation, indicated by T cell infiltration and activated microglia. Simultaneously, Lewy bodies were detected in the brains of rhesus macaques, confirming SARS-CoV-2′s ability to induce neuroinflammation alongside α-synucleinopathy [100]. Moreover, there is a case report of a patient who developed catatonia symptoms, i.e., sub-stupor, immobility, catalepsy, and rejection following COVID-19 infection and was finally diagnosed with DLB instead of delirium [155]. Catatonia is a state associated with several disorders including autism spectrum disorders, schizophrenia, and mood disturbances like depression. It is strongly influenced by neuroinflammation, which plays a crucial role in the development and progression of these conditions [162]. A growing number of COVID-19 patients have also reported experiencing catatonia [163]. Catatonia is often confused with delirium, which presents similar clinical symptoms. In the DLB case above, catatonia appeared for the first time after SARS-CoV-2 infection and did not respond to lorazepam, though electroconvulsive therapy (ECT) provided relief. Diagnostic imaging, including a dopamine transporter (DAT) scan and ^123^I-meta-iodobenzylguanidine imaging, showed reduced uptake, leading to a diagnosis of DLB rather than delirium [155].

Although the lockdown strategies implemented by many countries effectively limited SARS-CoV-2 transmission, this period proved to be highly challenging for mental health, particularly for vulnerable elderly individuals with dementia. Several reports have indicated a deterioration of cognitive and neuropsychiatric symptoms in patients with DLB during lockdown [164,165]. It was observed that altered subjective perceptions of the passage of time among DLB patients occurred alongside worsening disease features reported by their caregivers during this period [164]. Another study suggested that strict lockdowns lasting at least six months were associated with accelerated declines in cognitive function and neuropsychiatric symptoms in both DLB and AD patients. Among individuals with mild cognitive impairment (MCI), AD, and DLB, those with DLB showed a greater decline in Mini-Mental Status Exam (MMSE) scores, with more than half experiencing worsening neuropsychiatric inventory scores. Additionally, DLB patients exhibited a faster decline in MMSE scores compared with those with AD [165]. According to the concept of social health, the reduction in daily physical activity and social interaction for patients with dementia may significantly impact their long-term functioning. This consideration may have informed recommendations for dementia patients and their caregivers during the COVID-19 pandemic [166,167]. While potential links between SARS-CoV-2 and DLB are suggested, more research is needed to establish a clear relationship.

### 3.2. MSA

In MSA, α-syn aggregates are primarily found within oligodendrocytes such as Papp–Lantos inclusions. There are two variants of MSA: the first, characterized by striatonigral degeneration, presents symptoms similar to PD (MSA-P). In contrast, MSA with cerebellar symptoms affecting balance and coordination (MSA-C) results from olivopontocerebellar atrophy [168,169]. Recently there was reported a case of a patient hospitalized for COVID-19 who subsequently developed ataxia, progressive dizziness, and blurry vision, leading to a diagnosis of MSA-P [156]. This observation indirectly supports a causal relationship between coronavirus infection and the development of α-synucleinopathy. The etiology of MSA is complex and includes genetic and epigenetic factors, α-synuclein pathology, disrupted iron homeostasis, neuroinflammation, and mitochondrial dysfunction [170]. Among these, neuroinflammation is the most well documented and likely the missing link between SARS-CoV-2 infection and the onset of MSA. In an autopsy study of individuals who had COVID-19, the majority exhibited astrogliosis, microgliosis, and infiltration of cytotoxic T lymphocytes (CTL), particularly in the brainstem and cerebellum, along with meningeal CTL infiltration [171]. MSA is a common cause of cerebellar ataxia in adults [172]. In the case mentioned above, magnetic resonance imaging (MRI) showed moderate cerebellar and pontine volume loss with crossed hyperintensity of the pons, known as the "hot cross buns sign". The patient responded positively to treatment with amantadine and carbidopa/levodopa, as well as vestibular rehabilitation and meclizine. Given the close temporal link between SARS-CoV-2 infection and the emergence of MSA features, the authors suggest that this case may be connected to the COVID-19 infection, potentially triggering or accelerating pre-existing MSA [156]. Additionally, another case was reported of a patient with atypical parkinsonian features, including a lack of response to dopaminergic medication, early cervical dystonia, postural instability with falls, and dysautonomia following a prolonged SARS-CoV-2 infection. This patient met the criteria for a diagnosis of clinically probable MSA [173]. Similarly, as with other α-synucleinopathies, more research is needed to establish a clear causal relationship between MSA and COVID-19.

### 3.3. PAF

PAF, also known as idiopathic orthostatic hypotension or Bradbury–Eggleston syndrome, is an uncommon sporadic disorder that affects the sympathetic branch of the autonomous nervous system, without other neurological deficits. In PAF, α-syn pathology as typical and atypical LBs arises in SN, locus ceruleus, substantia innominata, as well as autonomic ganglia and nerves [174]. The degeneration of the sympathetic component of the autonomic nervous system (ANS) in PAF directs orthostatic hypotension, standing as the diagnostic criterium of PAF, and other symptoms such as genitourinary and intestinal dysfunction and sweating disorders [175]. The ANS complications, including hypertension, arrhythmias, and fatigue, are also common elements of post-COVID-19 cardiovascular syndrome [176]. Interestingly, several symptoms characterizing Long-COVID-19 recapitulate Postural Orthostatic Tachycardia Syndrome (POTS), which represents a frequent form of dysautonomia [177]. In POTS and PAF, an inverse dependence between the autonomic symptoms and cardiovascular changes affected by orthostatic stimulus intensity and the work ability index value was reported [178,179]. Notably, in patients who developed Long-COVID-19 autonomic syndrome, this association was more powerful. In line with these observations, SARS-CoV-2 may affect ANS and last for several months, leading to chronic autonomic syndrome [180].

### 3.4. RBD

RBD, characterized by the loss of normal muscle atonia during REM sleep, is associated with early α-syn alterations and is a well-documented prodromal marker of PD, often preceding the onset of typical motor symptoms by several years [181,182]. RBD is a sleep disorder that falls under the parasomnia category and is characterized by abnormal behaviors during sleep, such as gestures, vocalizations, movements, nightmares, and the loss of normal skeletal muscle atonia during REM sleep. The movements that occur in place of REM atonia can be complex, including actions like kicking or fighting in response to vivid and often frightening dream scenarios [183]. Based on reports of COVID-19 patients exhibiting RBD features, it is suggested that SARS-CoV-2 might potentially trigger this condition. For example, Heidbreder et al. (2021) found that more than one-third of COVID-19 patients analyzed using polysomnography showed REM sleep without atonia (RWA), a recognized neurophysiological substrate of RBD [157]. In the case–control study, isolated RWA (without clinical RBD) was observed more frequently in COVID-19 patients than in negative controls, indicating a potential link between prior COVID-19 infection and disturbances in the brainstem regions of the dorsal pons and/or ventromedial medulla [158]. Interestingly, dream-enactment behaviors became more common in the general population during the COVID-19 pandemic, with a notable increase among COVID-19 patients. Furthermore, in COVID-19-positive individuals, weekly dream-enactment behaviors, as measured by the RBD Single-Question Screen (RBD1Q), were positively associated with the severity of COVID-19 [184].

## 4. Age and Gender Aspects of Relationships Between SARS-CoV-2 Infection and α-Synucleinopathies

To date, no studies have examined the impact of COVID-19 infection on the development or progression of α-synucleinopathies concerning age or gender. Additionally, there are no data on how age or gender might influence α-syn’s role in COVID-19 progression. Consequently, any hypotheses regarding possible bidirectional relationships between SARS-CoV-2 infection and the α-synucleinopathies must rely on existing knowledge about COVID-19’s gender-specific effects and the influence of age on α-synucleinopathies.

Several studies, however, have investigated the relationship between age and COVID-19 severity. Data from China show a case fatality rate (CFR) below 0.4% for individuals aged 40 or younger, increasing to 1.3% at age 50, 3.6% at 60, 8% at 70, and 14.8% for those 80 and older [13,185]. Similarly, Italian data report CFRs below 0.4% for those under 40, rising to 1% at 50, 3.5% at 60, 12.8% at 70, and 20.2% at 80 [186]. The link between older age and more severe COVID-19 outcomes is often attributed to the generally poorer health and presence of comorbidities in older individuals, key factors in disease severity and progression.

Additionally, age-related immune decline and dysregulation are thought to heighten susceptibility to severe COVID-19 effects in this group [187]. Age-related changes in the immune system, known as immunosenescence, are believed to be a major cause of increased susceptibility to infections—especially respiratory infections like influenza—as well as a reduced immune response to vaccinations [188,189]. A key factor in the weakened antiviral immunity and increased susceptibility to SARS-CoV-2 in older adults may be the diminished IFN-I response, which contributes to greater viral replication—a mechanism also observed with influenza in cell cultures [190,191].

Studies have shown that older adults exhibit an impaired IFN-I response to influenza vaccination [192], and aging is associated with weakened CD4+ and CD8+ T cell responses in COVID-19. Declines in de novo T cell reactivity are considered a potential factor in age-related susceptibility to COVID-19 [193,194,195]. Lin et al. (2020) proposed a hypothesis regarding the role of immune and inflammatory factors in the dysregulation of the coagulation system in COVID-19 pathogenesis [196]. Research suggests a link between complement activation and endothelial dysfunction, which may contribute to microvascular thrombosis and multi-organ failure in severe cases [197,198,199]. However, few studies have examined aging in this context.

Inflammation is well documented and may arise from the senescence-associated secretory phenotype (SASP), which entails the copious secretion of pro-inflammatory signals in the tissue microenvironment and contributes to age-related conditions, persistent viral infections like cytomegalovirus (CMV), and other sources [200,201,202]. This pro-inflammatory environment may amplify inflammatory responses following SARS-CoV-2 infection, exacerbating cytokine storms in older adults and potentially affecting ACE2 expression, thereby facilitating viral penetration [203]. Enhanced cytokine secretion may impact the central nervous system, as SARS-CoV-2 has been shown to activate mast cells and microglia upon reaching the hypothalamus. This activation triggers the release of various pro-inflammatory cytokines and chemokines, including IL-1β, IL-6, IL-8, IL-33, CCL2, and TNF, which may contribute to the development or worsening of α-synucleinopathies.

Evidence suggests inflammation and microglial activation in graft deposits long before the accumulation of α-syn pathology in implanted dopaminergic neurons in patients with PD [204,205].

Pro-inflammatory cytokines such as TNF-α and IL-1β have been shown to facilitate the cell-to-cell propagation of α-syn in vitro, while TNF-α has also been implicated in promoting neuronal senescence, enhancing the SASP of neurons and subsequent lysosome-dependent secretion of α-syn [206]. Conversely, oligomeric α-syn released by neurons can stimulate microglial inflammatory responses through Toll-like receptor 2 (TLR-2) activation [207]. This evidence has led to a proposed model emphasizing the role of an inflammatory microenvironment in the spread of aggregates: initial protein aggregation induces a chronic inflammatory state, which in turn fosters a microenvironment conducive to further aggregation in neurons. This establishes a self-perpetuating cycle between protein aggregation and inflammation [208].

Another potential mechanism through which SARS-CoV-2 may exert detrimental effects on the CNS—likely influenced by aging—is mitochondrial damage. Mitochondria are critical to the pathophysiology of age-related disorders, including PD and other α-synucleinopathies [209]. The relationship between aging and mitochondrial dysfunction is profound, encompassing alterations in mitochondrial biogenesis and dynamics, increased oxidative stress, and impaired energy metabolism. These mitochondrial disturbances significantly contribute to the development of various age-related conditions and the aging process itself. Age-related mitochondrial dysfunction may play a crucial role in the oligomerization, aggregation, and toxicity of α-syn. SARS-CoV-2 infection may exacerbate mitochondrial damage due to NF-κB-mediated inflammatory responses [210,211,212]. Based on these findings, it can be hypothesized that older individuals are more susceptible to α-syn toxicity and neurodegeneration following SARS-CoV-2 infection. SARS-CoV-2 infection through various mechanisms, especially those related to cytokine storm, inflammation, mitochondrial damage, or oxidative stress, may lead to α-synucleinopathy and neurodegeneration in this vulnerable population.

Regarding gender-related aspects, published research suggests that the relationship between α-synucleinopathies and SARS-CoV-2 infection may be complex and influenced by age and hormonal status. Evidence indicates that the two primary sex steroid hormones inversely regulate ACE2 expression: estrogen downregulates the main SARS-CoV-2 receptor in various organs, while testosterone upregulates it [213,214]. Studies show that plasma ACE2 levels are higher in men than in women. Additionally, androgens stimulate TMPRSS2 expression in human lung epithelial cells, whereas their absence inhibits this expression [215]. TMPRSS2 is known to proteolytically activate several influenza viruses, coronaviruses, and SARS-CoV-2 [216]. Men with higher TMPRSS2 expression are more susceptible to infections from SARS-CoV [216] and MERS-CoV [217]. Therefore, the modulation of TMPRSS2 by testosterone may help explain the male predominance observed in these infections [218].

Testosterone may not only increase men’s susceptibility to SARS-CoV-2 infection but also weaken antiviral immunity. Several studies [219] confirm that testosterone reduces immune responses in men. By suppressing T helper (Th) 2 and Th17 cells, testosterone decreases antibody production and impairs B cell proliferation, thus compromising adaptive immunity [220]. In dendritic cells, testosterone reduces interleukin levels (IL-10, IL-13, and IL-4) and lowers the expression of the MHC-II receptor on antigen-presenting cells [221]. These effects are linked to delayed antibody responses in severe COVID-19 cases and lower IgG production in men compared with women, which may correlate with worse prognoses [222]. Interestingly, testosterone also increases anti-inflammatory cytokine levels in vivo, challenging the idea that it solely suppresses inflammation. It has been shown to enhance IL-10 expression while decreasing levels of IL-6, IL-1, and TNF-α [223]. Thus, at younger ages, elevated testosterone levels may render men more susceptible to SARS-CoV-2 infection and weaken their antiviral immunity, potentially leading to more severe infections compared with women. Simultaneously, the reduced inflammatory response in men may protect them from cytokine storms and associated complications.

In turn, estradiol has been shown to reduce ACE2 levels in the kidneys of women, while ovariectomy, which removes estrogen, increases ACE2 activity and expression in kidneys and adipose tissue in mice [213]. Additionally, recent studies suggest that increased estrogens can also enhance antiviral immunity [224]. Estradiol stimulates the production of interferon type 1 by T lymphocytes after binding to ER [225]. Increased levels of interferon I and III are associated with a milder course of COVID-19 [226]. Additionally, estrogen increases the expression of T cell chemokine receptor 5 (CCR5) and influences the increased adhesion of blood lymphocytes to endothelial cells [218,227], increases the production of IL-4 and the development of T helper type 2 cells [228] as well as stimulates T helper cell type 1 differentiation by reducing T helper cell type 17 and IL-17 cytokines [229].

An additional hypothesis explains the superior immunity of females against SARS-CoV-2 infection, linked to their double dose of the X chromosome, which houses several immunity-related genes such as Fork-head box P3 (FOXP3), CD40 ligand (CD40L), Toll-like receptor 7 (TLR-7), and Toll-like receptor 8 (TLR-8) [230]. Although X chromosome inactivation occurs during female embryogenesis, some proteins encoded on the X chromosome can still be produced in a biallelic manner. This biallelic expression of X-linked genes in immune cells may lead to heightened immunity or unfavorable inflammatory responses [231]. One key protein is TLR-7, crucial for pathogen recognition and the activation of innate immunity and predominantly found in monocytes, plasmacytoid dendritic cells (PDCs), and B cells [232]. Women may exhibit overexpression of TLR-7 due to biallelic expression compared with men [233,234]. Consequently, this increased expression may enhance women’s immune response, offering a protective advantage against COVID-19 infection and its severe outcomes. This protection may persist even after menopause, despite the decline in estrogen synthesis.

Results from various studies involving humans and rodents indicate that estrogen may provide protection to females against cytokine storms and inflammation. At physiologically elevated levels, estrogen appears to inhibit the production of several pro-inflammatory cytokines, such as IL-6, IFN-α, IL-1, and chemokine (C-C motif) ligand 2 (CCL2), from monocytes and macrophages. This action helps to prevent the migration of neutrophils and monocytes to inflammatory sites [235].

The data suggest that younger men may be at a higher risk of COVID-19 infection and severe illness compared with women. In this age group, both men and women appear to have a lower risk of developing a cytokine storm, a factor that could otherwise promote α-synuclein aggregation. This protective effect may stem from testosterone and estradiol, which help to reduce the production of pro-inflammatory cytokines, including IL-1, IL-6, and TNF-α [236].

However, these dynamics may shift with age as sex hormone levels decline. Among older adults, both men and women may experience similar risks of SARS-CoV-2 infection, cytokine storm development, and related complications affecting the central nervous system. The interplay between sex, SARS-CoV-2 infection, and α-synucleinopathy is thus complex, shaped by age and hormonal status. Further research is needed to clarify these relationships and their broader implications.

## 5. Conclusions

The emerging evidence suggests that COVID-19 may have lasting effects on the nervous system and brain function, with a potential link between α-synucleinopathies and SARS-CoV-2 infection. However, the nature of this relationship and its direct causality remain poorly understood. Several mechanisms could explain how SARS-CoV-2 infection might contribute to the onset or progression of neurodegenerative diseases such as PD and other α-synucleinopathies, involving both direct and indirect alterations of α-syn.

These mechanisms include the viral-induced dysfunction of α-syn resulting from direct protein–protein interactions that accelerate the conversion of α-syn into pathological multimeric forms, such as oligomers and protofibrils, promoting its spread and leading to widespread neurodegeneration. Additionally, chronic neuroinflammation triggered by SARS-CoV-2 may cause pathological changes in α-syn, further supporting a potential connection between these disorders.

Interestingly, in the context of SARS-CoV-2 infection, α-syn may play a “Janus-faced” role, potentially acting protectively through upregulation as part of the immune response to viral infections. Understanding the interplay between SARS-CoV-2 infection and the dual nature of α-syn is crucial for managing and treating patients with pre-existing neurological conditions who contract COVID-19, as well as for exploring the role of long COVID in the development of α-synucleinopathies.

To date, several potential therapies have been proposed, targeting pro-inflammatory processes, ACE2 receptors, ERK1/2 activity, and intracellular Ca^2+^ homeostasis (Table 3). However, the search for effective treatments for long COVID is ongoing, necessitating more in-depth studies and subsequent clinical trials to identify promising therapeutic targets and develop successful interventions.

## Figures and Tables

**Table 2:** The prevalence, outcomes, and prognosis of COVID-19 in patients diagnosed with PD.

Aim	Method/Subject of Analysis	Results	Reference
Meta-analysis aimed to determine the prevalence of COVID-19, its symptoms in elderly patients with PD and the association between PD and COVID-19.	Twenty articles were selected from January 2019 to 20 October 2021.	The prevalence of COVID-19 and the hospitalization of patients with PD was 1.06% and 0.98%, respectively; the prevalence of depression and anxiety during the pandemic in these groups was 46% and 43%, respectively; the risk of COVID-19 infection was equal in the PD patients and healthy controls.	[69]
Analysis of the prevalence of neurological disorders (including PD) in COVID-19 without overlapping meta-analysis errors.	Four meta-analyses involving 182,386 COVID-19 patients, published from November 2019 to September 2021.	The combined prevalence of PD during the COVID-19 pandemic was 0.67%; PD was not a statistically significant risk of mortality in COVID-19 patients (Odds Ratio [OR] = 3.94).	[70]
Review of all qualified studies to quantify the strength of affinities between pre-existing neurodegenerative diseases, SARS-CoV-2 vulnerability, and COVID-19 outcome.	Pre-registered systematic review with frequentist and Bayesian meta-analyses; 9 January 2023 was the final search date; 136 primary studies (total sample size n = 97,643,494), reporting on 268 effect-size estimates, met the inclusion criteria.	The odds for a positive SARS-CoV-2 test result were raised for individuals with pre-existing AD (OR = 2.86), dementia (OR = 1.83), and PD (OR = 1.65). People with pre-existing AD were at a higher risk for COVID-19-related hospital admission (OR = 3.72), but people with MCI, PD, or mixed dementia were not. People with AD and PD were at a higher risk for COVID-19-related intensive care unit admissions (pooled OR range: 1.55–1.65). All neurodegenerative disorders were at a higher risk for COVID-19-related mortality (pooled OR range: 1.56–2.27). In general, people with neurodegenerative disease and MCI are at a disproportionally high risk of acquiring COVID-19 and have a poor outcome once infected.	[71]
The assessment of the association between pre-existing neurological conditions and COVID-19 outcomes.	Literature review of systematic reviews, meta-analyses, and scoping reviews published between 1 January 2020 and 1 January 2023. Thirty-nine articles fulfilled the inclusion criteria, with data estimating >3 million people from 51 countries.	In total, 92.3% of the articles suggested a significant link between pre-existing neurological disorders including cerebrovascular disease, PD, AD and other dementias, and epilepsy and an increased risk of severe COVID-19 and mortality in the acute infectious period.	[72]
The systematic review and meta-analysis aimed to investigate the influence of the COVID-19 pandemic on neuropsychiatric disorders (depression, anxiety, stress) and sleep disturbances (sleep quality, insomnia), as well as the quality of life among patients with PD, MS, and AD compared with healthy people.	Observational studies (i.e., cross-sectional, case–control, cohort) raised from the research of 7 databases between March 2020 and December 2022. An analysis of eighteen studies (PD = 7, MS = 11) with a total of 627 individuals with PD (healthy controls = 857) and 3923 individuals with MS (healthy controls = 2432); twelve studies (PD = 4, MS = 8) were included in the meta-analysis.	The COVID-19 pandemic negatively affected people with PD, evidenced by significantly higher levels of depression and stress, measured by standardized mean differences (SMD) = 0.40 and 0.60, respectively. MS patients also presented higher levels of depression/stress, and additionally lower quality of life compared with the healthy control groups.	[73]
Meta-analysis of factors that affect the well-being of PD individuals from diverse populations during the pandemic.	Research of articles published between 2020 and 2022; the analysis includes twenty-seven studies involving 13,878 patients from America, Europe, Asia, and Africa.	High prevalence of diminished physical activity and exercise, and aggravating motor and neuropsychiatric symptoms (17–56%) during the COVID-19 pandemic, with patients in lower-income countries being exceptionally vulnerable, i.e., anxiety (adjusted Odds Ratios, [aOR] = 8.94), sleep (aOR = 5.16), and PD symptoms (aOR = 3.57). Younger age correlated with decreased physical activity, exercise, sleep, and worsening PD symptoms. Female PD patients reported a more pronounced decrease in physical activity and sleep disturbances.	[74]
Systematic review and meta-analysis aimed to determine the pooled prevalence of COVID-19 in PD patients.	Thirty articles for meta-analysis with the number of included patients differed between 10 and 64,434; published before Sep 2021.	The pooled prevalence of COVID-19 infection in PD cases was 5% besides mortality and hospitalization rates were 12% and 49%, respectively. The pooled prevalence of fever and cough in cases with PD was 4% and 3%, respectively.	[75]
Systematic review aimed to determine the impact of PD on the COVID-19 prevalence and patient prognosis.	Thirteen papers including 8649 PD patients and 88,710 control subjects/till 12 March 2021.	The pooled prevalence rate of COVID-19 among PD patients was 2.12%. The hospitalization rate for PD patients with COVID-19 was 39.89%, while the total mortality rate was 25.1%. There were no significant differences in hospitalization and mortality rates among COVID-19 patients with and those without PD. Fever, cough, fatigue, and anorexia existed as the most common manifestations with rates of 72.72%, 66.99%, 61.58%, and 52.55%, respectively.	[68]
Systematic review aimed to determine the influence of factors connected with COVID-19 in PD patients.	Literature research up to November 2020 (updated until 1 April 2021); finally, six studies (four case–control studies and two cross-sectional studies) in the qualitative and quantitative syntheses.	The following factors were connected with COVID-19 in PD patients: obesity (OR: 1.79) and pulmonary disease (OR: 1.92), COVID-19 contact (OR: 41.77), vitamin D supplementation (OR: 0.50), hospitalization (OR: 11.78), and death (OR: 11.23). The authors did not find any significant correlation between COVID-19 and hypertension, diabetes, cardiopathy, cancer, any cognitive problem, dementia, chronic obstructive pulmonary disease, renal or hepatic disease, smoking, and tremor.	[76]
Analysis of the relationship between PD and in-hospital outcomes of COVID-19.	A total of 12 studies with 103,874 COVID-19 patients.	PD was connected with poor in-hospital outcomes, OR = 2.64. Subgroup analysis showed that PD was connected with severe COVID-19 OR = 2.61, and mortality from COVID-19 Relative Risk (RR) = 2.63. Meta-regression showed that the association between PD and in-hospital outcomes of COVID-19 was influenced by age, but not by gender, dementia, hypertension, and diabetes.	[77]

**Table 3:** Potentially shared targets for both PD and COVID-19.

Molecular Target	Example of Drug	Reference
Inhibition of NLRP3-dependent programed cell death, called pyroptosis and autophagy regulation, promotion of α-syn clearance, and restoration of proteasome 20 S activity. Reduction in α-syn (Ser129) phosphorylation.	Salidroside	[113,114,115]
Blocking of the P2X7R/NLRP3 axis triggering a cytokine storm. Reduction in ATP concentration and the activation of IL-1β and IL-6. Prevention of the influx of Ca^2+^ and the occasion of α-syn mutations.	Lidocaine	[119,150]
Counteraction glutamine-mediated excitotoxicity by inhibition of calcium influx into the cells by NMDA receptor channel blocking. Inhibition of SARS-CoV-2 viral channel activity, like the Protein E (envelope) cation channel, representing SARS-CoV-2 viroporin involved in its virulence.	Amantadine, Memantine	[131,151]
Regulation of calcium homeostasis by mGluR5.	Lithium	[138,139,140]
Switch microglia to anti-inflammatory and neuroprotective M2-phenotype. TLR-4 antagonism, ameliorating cytokine storm. Disruption of SARS-CoV-2 S protein binding to ACE2. Reduction in the phosphorylation/activity of ERK1/2.	Low-Dose Naltrexone	[141,142,143]
Prevention of the Fenton reaction and the ferroptosis inhibition.	Deferoxamine, Phyto-chelators like Caffeic acid, Curcumin, α-Lipoic acid (ALA), and Phytic acid	[144,146,147,148]
Scavenging of lipid peroxides and prevention of oxidative damage by lipophilic antioxidants.	Ferrostatin-1, Liproxstatin-1	[144,149]
Anti-oxidative effects on the DA neurons in PD by increasing Nrf2 expression, and inhibition of the interaction of ACE2 with the S protein of SARS-CoV-2.	Flavones (Chrysin, Quercetin)	[152,153,154]

**Table 4:** α-Synucleinopathy diagnosis after COVID-19. The case of patients.

Disease	Case of Patient Description	Ref.
**DLB**	A 68-year-old man after COVID-19 with catatonia symptoms, like sub-stupor, immobility, catalepsy, and rejection. Catatonia appeared for the first time after SARS-CoV-2 infection and did not respond to lorazepam, though the ECT provided relief. Diagnostic imaging, including a DAT scan and ^123^I-meta-iodobenzylguanidine imaging, showed reduced uptake, leading to the final diagnosis of DLB instead of delirium.	[155]
**MSA**	A 65-year-old woman hospitalized for COVID-19 developed ataxia, progressive dizziness, and blurry vision. The evaluation noted rightward nystagmus, resting tremor of the right hand, slowed finger tapping bilaterally, right dysmetria, and a shuffling gait. Lumbar puncture was negative. Brain MRI indicated moderate cerebellar and pontine volume loss with crossed hyperintensity of the pons, known as the “hot cross buns sign”. Videonystagmography proved the cerebellar etiology of her symptoms. MSA with parkinsonian components (MSA-P) was finally diagnosed. The patient reacted positively to therapy with amantadine and carbidopa/levodopa, as well as vestibular rehabilitation and meclizine. Given the close temporal link between SARS-CoV-2 infection and the emergence of MSA features, the authors suggest that this MSA-P case may be related to the COVID-19 infection.	[156]
**RBD**	Patients with suspected sleep disorders after acute COVID-19 underwent video-polysomnography (v-PSG). At 60 days post-diagnosis, 4/11 patients (36%) were diagnosed with obstructive sleep apnea (OSA). Also, 4/11 patients showed REM sleep without atonia (RWA), a recognized prodromal stage of RBD, and two additional patients showed an RWA index within the highest range of normality.	[157]
	The results of the case–control studies of 25 patients with previous COVID-19 infection compared with 25 age–sex matched controls who tested negative for COVID-19 before polysomnography. Isolated RWA occurred more frequently in the COVID-19 (9/25 patients, 36%) patients than in the controls (3/25 patients, 12%).	[158]
